# Beyond Uterine Natural Killer Cell Numbers in Unexplained Recurrent Pregnancy Loss: Combined Analysis of CD45, CD56, CD16, CD57, and CD138

**DOI:** 10.3390/diagnostics10090650

**Published:** 2020-08-29

**Authors:** Maia Chiokadze, Christin Bär, Jana Pastuschek, Boris V. Dons’koi, Kseniia G. Khazhylenko, Ekkehard Schleußner, Udo R. Markert, Rodolfo R. Favaro

**Affiliations:** 1 Placenta Lab, Department of Obstetrics, Jena University Hospital, 07747 Jena, Germany; mchiokadze2017@gmail.com (M.C.); christin.baer@med.uni-jena.de (C.B.); jana.pastuschek@med.uni-jena.de (J.P.); ekkehard.schleussner@med.uni-jena.de (E.S.); rodolfo.favaro@med.uni-jena.de (R.R.F.); 2 The Center for Reproductive Medicine “Universe”, 0159 Tbilisi, Georgia; 3 Laboratory of Immunology, Institute of Pediatrics, Obstetrics and Gynecology, National Academy of Medical Sciences of Ukraine, 04050 Kyiv, Ukraine; boris_donskoy@ukr.net; 4 Reproductive Medicine Clinic Isida, 02000 Kyiv, Ukraine; k_khazhylenko@isida.ua

**Keywords:** human, endometrium, recurrent pregnancy loss, natural killer cells, CD16, CD45, CD56, CD57, CD138

## Abstract

Changes in the number and cytotoxic potential of uterine Natural Killer (uNK) cells have been associated with reduced fertility. To provide a better characterization of immunophenotypes in the endometrium of women with uRPL (unexplained recurrent pregnancy loss), we examined the applicability of a set of five immune cell markers. The concentration (cells/mm^2^) of CD45^+^ leukocytes, CD56^+^ uNK cells, and CD138^+^ plasma cells as well as of CD16^+^ and CD57^+^ cells, which indicate high cytotoxic uNK cells, were assessed by immunohistochemistry in endometrial biopsies from 61 uRPL patients and 10 controls. Control fertile endometria presented 90–300 CD56^+^ uNK cells/mm^2^. uRPL cases were classified in subgroups of low (uRPL-CD56^low^ < 90 cells/mm^2^), normal (uRPL-CD56^normal^ 90–300 cells/mm^2^), and high uNK cell counts (uRPL-CD56^high^ > 300 cells/mm^2^). Some cases from the uRPL-CD56^low^ and uRPL-CD56^normal^ subgroups showed elevated proportions of cytotoxic CD16^+^ and CD57^+^ cells in relation to CD56^+^ cells. In the uRPL-CD56^high^ subgroup, the CD57/CD56 ratio was reduced in most samples and the CD16/CD56 ratio was unaltered. Analysis of CD138 excluded the influence of chronic endometritis on these observations. Our results reinforce a link between uRPL and a dysfunctional endometrial environment associated with distinct immune cell profiles.

## 1. Introduction

The demise of two or more subsequent clinical pregnancies until the 24th week fulfills the latest strict criteria proposed for diagnosis of recurrent pregnancy loss (RPL) [1,2,3]. RPL poses a series of challenges to reproductive medicine worldwide and constitutes a substantial economic burden. Furthermore, it represents a highly frustrating life event for many couples, and its repetitive nature intensifies the psychological distress experienced [4].

RPL affects 1–5% of women of reproductive age. Documented causes of this multifactorial, heterogeneous disorder include advanced maternal age, parental chromosomal abnormalities, uterine anomalies, hormonal and metabolic disturbances, and acquired thrombophilia [2,5,6]. Despite intense research, more than 50% of cases remain unexplained (uRPL). Many are attributed to immune factors [7,8]. Disturbed endometrial immunoreactivity due to inadequate number and function of endometrial immune cell populations has been associated with uRPL [9,10,11,12].

A sophisticated and fine-tuned set of immune interactions takes place in the endometrium to support embryo development and to prevent its rejection [13,14,15]. Around 40% of the endometrial stromal cells are composed of leukocytes. Uterine Natural Killer (uNK) cells usually are the most abundant, reaching up to around 80% of the endometrial immune cell population [13]. The number of uNK cells varies through the menstrual cycle, marked by a dramatic increase in the mid-luteal phase [11,16,17]. uNK cells remain in the decidua until the end of pregnancy, when their number decreases [18].

Natural Killer (NK) cells have acquired specific functions associated with the demands of the compartments in which they settle. The endometrium has a peculiar population of NK cells designed to cope with pregnancy demands. These cells are pivotal in the maintenance of endometrial homeostasis and maternofetal tolerance [19,20,21,22]. They contribute to the process of decidualization [23] and, driven by IL-15, selectively target and clear senescent decidual cells as part of the endometrial regeneration cycle [24]. Their abundance at the time of implantation and their presence in close proximity to invading extravillous trophoblast cells and blood vessels indicate their roles in human pregnancy. These observations are supported by studies performed in animal models and in vitro systems demonstrating that uNK cells contribute to modulation of trophoblast invasion, remodeling of uterine spiral arteries, and tolerogenic immune reactions in pregnancy [25,26,27,28,29,30].

In the human endometrium, there are two main populations of phenotypically and functionally distinct uNK cells. The predominant subset (approximately 90%) encompasses cells expressing the archetypal NK cell surface marker CD56 (neural cell adhesion molecule (NCAM)), a member of the immunoglobulin superfamily) at a high density. These cells lack CD16 (FcRIII, a low-affinity receptor for IgG complexes), which is, in contrast, highly expressed in cytotoxic peripheral blood NK cells. These CD56^bright^CD16^neg^ uNK cells have little cytotoxic activity and represent a rich source of different cytokines, growth factors, and angiogenic factors, whereas the minor CD56^dim^CD16^+^ subset has limited cytokine output and is primarily responsible for NK cell cytotoxicity [21,25,31,32].

Although not deterministic, a certain range of CD56^+^ uNK cells seems to support the establishment and maintenance of pregnancy. Either low or high amounts of uNK cells detected in RPL endometrium may be detrimental to the maternofetal interactions [9,10,12,33]. An imbalance between immunoregulatory and cytotoxic uNK cells has been described in uRPL. The expression of CD16, followed by CD57 (a terminally sulfated glycan carbohydrate), designates the last stages of NK cell differentiation and a robust cytotoxic potential. CD57 also denotes terminally differentiated senescent immune cells [34,35]. The number of both CD16^+^ and CD57^+^ cells are increased in the endometrium of women with recurrent implantation failure (RIF) and uRPL [10,11,36,37,38]. Impaired production of cytokines and deregulated cytotoxicity are proposed mechanisms by which uNK cells generate an endometrial environment incompatible with embryo development [7,39,40].

Chronic endometritis, identified by the presence of CD138^+^ plasma cells in the endometrium [41,42], constitutes another factor promoting unfavorable endometrial immune conditions due to abnormal levels of inflammatory cytokines and chemokines [43,44]. Besides, altered numbers of CD56^bright^ CD16^neg^ cells and higher CD3^+^ T lymphocytes were found in the secretory endometrium of infertile patients with chronic endometritis compared to those without it [45]. Decreased levels of macrophage inflammatory protein 1β (MIP-1β) may be involved in this process [46]. These alterations promoted by chronic endometritis have the potential to interfere with maternofetal interactions, leading to pregnancy failure [47]. 

In the present study, we sought to determine the applicability of a combination of different immune markers to deepen the characterization of immunophenotypes in endometrial biopsies from women with uRPL. The concentrations of leukocytes (CD45^+^), plasma cells (CD138^+^), and uNK cells (CD56^+^), as well as their cytotoxic competence (CD16^+^ and CD57^+^) were evaluated by immunohistochemistry in the endometrium of fertile controls and uRPL patients. The analysis of these markers dependent on uNK cell count ranges unveiled previously unappreciated features of endometrial immune cell populations in uRPL.

## 2. Materials and Methods

### 2.1. Human Endometrial Samples

Endometrial biopsies from fertile control and uRPL groups were collected in the mid-luteal phase of the menstrual cycle (from day 19 to 22) with the aid of a pipelle and were fixed in formalin for at least 24 h. Cases of uRPL (*n* = 61), with at least 2 consecutive pregnancy losses were selected from the endometrial tissue biobank at the Placenta-Lab, Jena University Hospital, Germany. Patients with concomitant identifiable causes of pregnancy loss were excluded. Control endometrial samples (*n* = 10) were collected before the beginning of the protocol for oocyte donation in women with at least one viable pregnancy and no miscarriages in anamnesis. They did not have any autoimmune disease, antibiotic therapy, hormonal treatment, or vaccination for at least 3 months before endometrial sampling. These women were recruited at the Laboratory of Immunology, Institute of Pediatrics, Obstetrics and Gynecology, National Academy of Medical Sciences of Ukraine, Kyiv, Ukraine. The mean age of the fertile controls and uRPL patients were 27 and 33.5 years, respectively.

The study was approved by the respective local ethic committees (Jena University Hospital, registration number 2019-1305 from 8 February 2019). All procedures were in accordance with ethical standards on human experimentation preconized by the Helsinki Declaration of 1964 and its later amendments. All patients gave written informed consent.

### 2.2. Immunohistochemical Staining of Immune Cell Markers

Paraffin-embedded endometrial biopsies from the control and uRPL groups were sectioned at 4 μm in a microtome and deposited on SuperFrost slides (Menzel, Germany). The investigated markers were assessed in subsequent sections. Following dewaxing in xylene and rehydration through descending ethanol concentrations, antigen retrieval was achieved in a citrate buffer at >95 °C for 15 min. Slides were washed in Tris-buffered saline-Tween20 0.05% (TBST). For inhibition of endogenous peroxidase activity, and tissue sections were incubated with peroxidase block solution (Dako, Germany) for 10 min and washed in TBST. Primary antibodies were prepared in antibody diluent solution (Dako, Germany) and incubated for 1 h at room temperature (RT). Antibody specifications and dilutions are shown in Table 1.

Following washing in TBST, the slides were incubated with the secondary antibody (labelled polymer-horseradish peroxidase (HRP) anti-mouse, clone: DAK-GO1, Dako, Germany) for 30 min at RT. The peroxidase reaction was developed with DAB (3,3′-diaminobenzidine; Dako, Germany) and discontinued with water after 15 min. Counterstaining with hematoxylin was followed by mounting of slides with histofluid and cover slip.

### 2.3. Cell Counting and Statistical Analysis

Slides were documented and analyzed using an Axio Imager A2 microscope and Zen Blue software (Zeiss, Germany). The first field to be captured was selected at random near the luminal epithelium. Five nonoverlapping fields (20× objective) were documented per case. A positive cell was defined as an immuno-positive structure associated with a hematoxylin-stained cell nucleus. Cells were counted manually by two investigators unaware of the identification of the groups. The cell count tool from FIJI/Image J software (National Institutes of Health) was used. Cells within the glandular epithelium, blood vessels, or areas filled with blood were not considered. Data were expressed as mean number of cells/mm^2^. The ratios between markers were calculated and expressed as percentages. Statistical analyses were performed using the GraphPad Prism software (Graphpad Software, Inc., San Diego, CA, USA). Mean values were evaluated by the nonparametric Mann–Whitney test. Correlations were determined by the Spearman correlation test, and frequency distributions were compared using the chi-square test. Values of *p* ≤ 0.05 were considered statistically significant.

## 3. Results

Immunolocalization of CD138^+^ plasma cells was carried out to detect the presence of chronic endometritis in the analyzed endometrial biopsies. In the control group, 20% presented <3 CD138^+^ plasma cells/10 mm^2^, whereas in 80%, these cells were absent. Similarly, 22% of uRPL endometria had <3 CD138^+^ plasma cells/10 mm^2^ and 78% were negative. Applying the criteria from Liu et al. (2018) [48], by which chronic endometritis is diagnosed by ≥5.15 CD138^+^ plasma cells/10 mm^2^, the potential influence of this condition in the results of the present study was excluded. 

**Figure 1:**
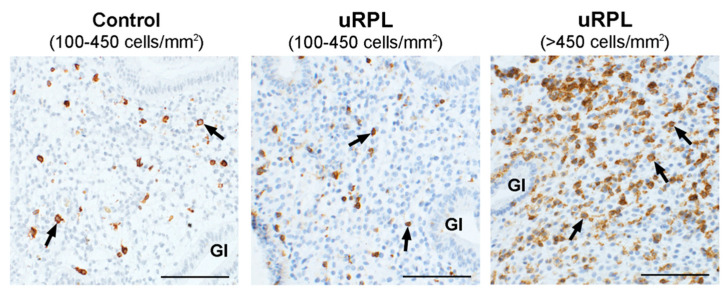
Immunohistochemical localization of CD45^+^ cells (arrows) in the endometrium of the control and unexplained recurrent pregnancy loss (uRPL) groups (100–450 cells/mm^2^) and of uRPL patients (100–450 and >450 cells/mm^2^). Gl: endometrial glands. Scale bar = 100 µm.

**Figure 2:**
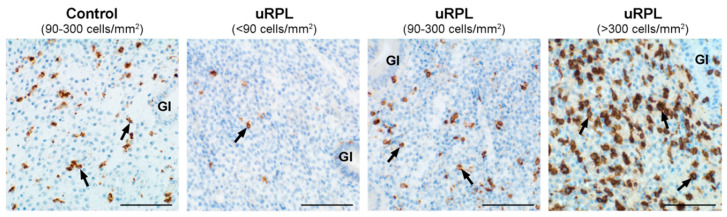
Immunohistochemical localization of CD56^+^ cells (arrows) in the endometrium of the control (90–300 cells/mm^2^), uRPL-CD56^Low^ (<90 cells/mm^2^), uRPL-CD56^Normal^ (90–300 cells/mm^2^), and uRPL-CD56^High^ subgroups (>300 cells/mm^2^). Gl: endometrial glands. Scale bar = 100 µm.

**Figure 3:**
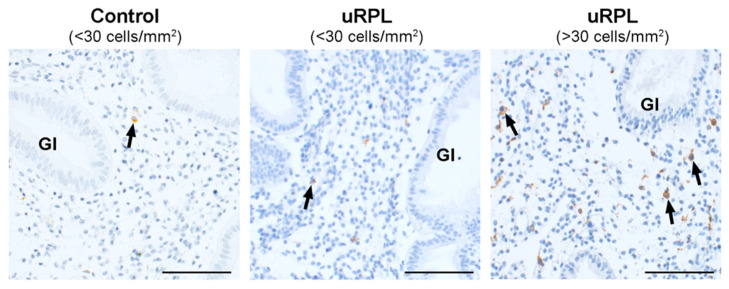
Immunohistochemical localization of CD16^+^ cells (arrows) in the endometrium of control (<30 cells/mm^2^) and uRPL cases (<30 and >30 cells/mm^2^). Gl: endometrial glands. Scale bar = 100 µm.

**Figure 4:**
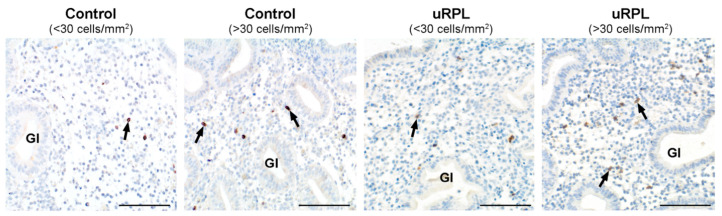
Immunohistochemical localization of CD57^+^ cells (arrows) in the endometrium of control (<30 and >30 cells/mm^2^) and uRPL cases (<30 and >30 cells/mm^2^). Gl: endometrial glands. Scale bar = 100 µm.

**Figure 5:**
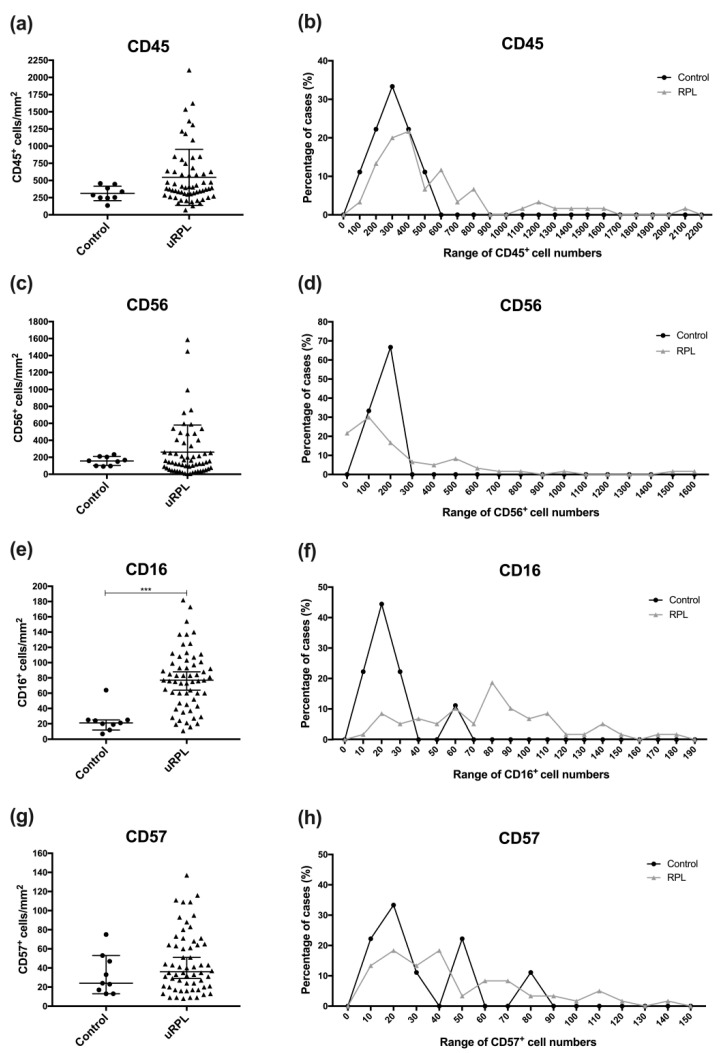
Mean values and frequency distribution of cell count ranges for CD45 (**a**,**b**), CD56 (**c**,**d**), CD16 (**e**,**f**), and CD57 (**g**,**h**) in the endometrium of controls (*n* = 10) and uRPL cases (*n* = 61): (**a**,**c**,**e**,**g**) Cell counts of all individual samples, their mean, and standard error and (**b**,**d**,**f**,**h**) the distribution of values from samples classified in ranges of 100 cells/mm^2^ (CD45 and CD56) or 10 cells/mm^2^ (CD16 and CD57). The relative frequency shows the percentage of samples in the respective range. Values are expressed in cells/mm^2^. *** *p* < 0.001, Mann–Whitney test.

**Figure 6:**
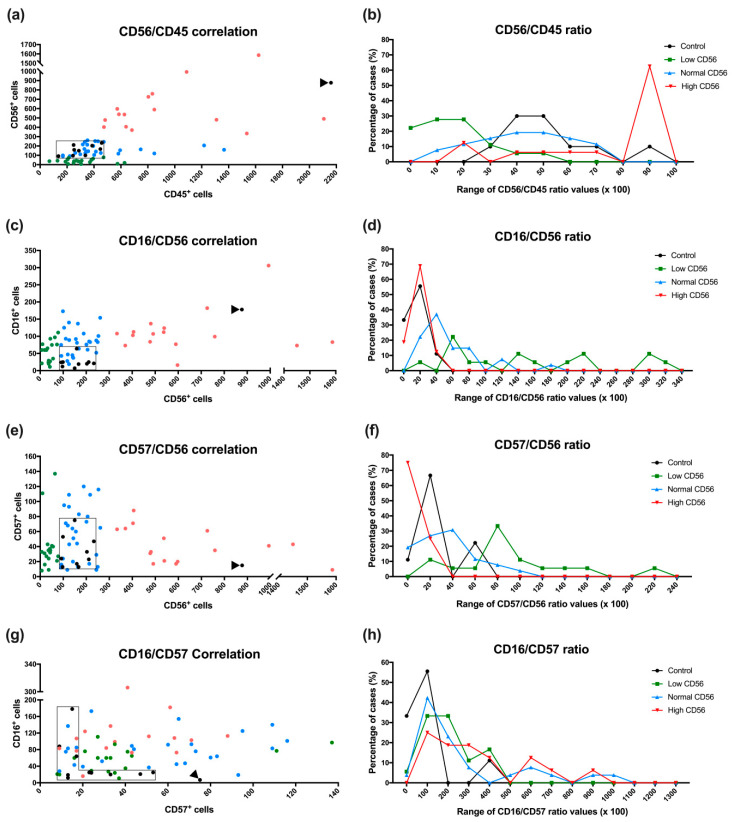
Correlation and ratio between CD56/CD45 (**a**,**b**), CD16/CD56 (**c**,**d**), CD57/CD56 (**e**,**f**), and CD57/CD16 (**g**,**h**) and distribution of their ratios (%) in the control (*n* = 10) and uRPL groups (*n* = 61): (**a**,**c**,**e**,**g**) each sample is represented by a dot (black: controls; green: uRPL-CD56/low; blue: uRPL-CD56/normal; and red: uRPL-CD56/high). Boxes surround the control values, and arrowheads show a single outlier control sample with signs of inflammation. (**b**,**d**,**f**,**h**) Samples are classified in stepwise ranges based on their ratios as indicated in the x axes.

Representative immunohistochemical staining of CD45, CD56, CD16, and CD57 in the endometrium from control and uRPL subgroups can be found in Figure 1, Figure 2, Figure 3 and Figure 4. The mean number of CD16^+^ cells was significantly increased in the endometrium of uRPL patients compared to controls (*p* < 0.001). No differences were observed in the mean values of CD45 (*p* = 0.06), CD56 (*p* = 0.99), and CD57 (*p* = 0.14) (Figure 5). Nevertheless, further analysis of these markers showed their different distributions in uRPL patients (*p* < 0.001 for CD45, CD56, and CD16; *p* = 0.003 for CD57) compared to controls (Figure 5). Following, a correlative evaluation of the investigated markers and their ratios demonstrated particular features depending on uNK cell count ranges (Figure 6). A detailed description of these results is presented below.

### 3.1. CD45

The concentration of CD45^+^ leukocytes in control endometria ranged from 100 to 450 cells/mm^2^. One sample in the control group showed an excessively high number of CD45^+^ leukocytes (2160 cells/mm^2^) and has been classified as inflamed since a high leukocyte number indicates an acute inflammatory process. Although present in the correlation graphs (Figure 6), values of this case were omitted from the calculations of control values. In uRPL patients, 65% of cases were within the control range (100–450 cells/mm^2^), whereas 35% showed >450 cells/mm^2^ (*p* < 0.001; Figure 1 and Figure 5).

### 3.2. CD56

In all control endometria, CD56^+^ uNK cells were present at densities between 90 to 300 cells/mm^2^. The inflamed control sample displayed an elevated uNK cell count (877 cells/mm^2^). Only 47% of uRPL cases displayed values similar to that of controls, 22% had low amounts (<90 cells/mm^2^), and 32% presented elevated numbers of CD56^+^ uNK cells (>300 cells/mm^2^) (*p* < 0.001). Based on these results, the uRPL patients were classified into subgroups with low (uRPL-CD56^low^ < 90 cells/mm^2^), normal (uRPL-CD56^normal^ 90–300 cells/mm^2^), and high uNK cell counts (uRPL-CD56^high^ > 300 cells/mm^2^) for further analysis of the data (Figure 2 and Figure 5).

### 3.3. CD16

Eight out of ten control endometria (89%) contained <30 CD16^+^ cells/mm^2^. One control sample (11%) showed 64 cells/mm^2^, and the inflamed one showed 178 CD16^+^ cells/mm^2^. In uRPL cases, 15% showed <30 cells/mm^2^ and 85% had >30 cells/mm^2^ (*p* < 0.001; Figure 3 and Figure 5). 

### 3.4. CD57

In the control group, 56% had <30 CD57^+^ cells/mm^2^ and 44% had >30 cells/mm^2^. uRPL patients presenting <30 cells/mm^2^ encompassed 32% of cases, whereas those with >30 cells/mm^2^ comprised 68% (*p* < 0.001; Figure 4 and Figure 5).

### 3.5. CD56/CD45 Correlation and Ratio

The concentration of CD45^+^ leukocytes in the control group was positively correlated with the number of CD56^+^ cells (*r* = 0.683; *p* < 0.01). In contrast, no correlations were detected for these markers in any uRPL subgroup: uRPL-CD56^low^ (*r* = 0.004; *p* = 0.99), uRPL-CD56^Normal^ (*r* = 0.3; *p* = 0.14), and uRPL-CD56^high^ (*r*= 0.239; *p* = 0.39).

Analysis of the CD56/CD45 ratio revealed that uNK cells accounted for around 30 to 70% of all endometrial leukocytes in eight out of nine control endometria. In one control sample, this value reached 85%. In the uRPL-CD56^Normal^ subgroup, 81% had similar CD56/CD45 ratios to those of controls whereas a small fraction (19%) presented high CD45^+^ cell counts and very low CD56/CD45 ratios (*p* = 0.5 vs. control). Differently, only 22% of cases from the uRPL-CD56^low^ subgroup had CD56/CD45 ratio values similar to that of controls. In the majority of the samples (78%), CD56/CD45 ratios were low, indicating that CD56^+^ uNK cells were greatly outnumbered by total CD45^+^ leukocytes (*p* < 0.001 vs. control). In the uRPL-CD56^high^ subgroup, 25% presented CD56/CD45 ratio values within the control range and 12% had extremely high numbers of CD45^+^ leukocytes and, hence, diminished CD56/CD45 ratios. Most endometria in the uRPL-CD56^high^ subgroup (63%), however, had high CD56/CD45 ratio values (*p* = 0.05 vs. control), showing that elevated CD45^+^ cell counts were due to excessive CD56^+^ uNK cells, which encompassed up to 95%. CD56/CD45 ratio values of the uRPL subgroups were different between each other (uRPL-CD56^low^ vs. uRPL-CD56^Normal^ *p* = 0.005; uRPL-CD56^low^ vs. uRPL-CD56^high^ groups *p* < 0.001; and uRPL-CD56^Normal^ vs. uRPL-CD56^high^ *p* = 0.002; Figure 6).

### 3.6. CD16/CD56 Correlation and Ratio

The number of CD16^+^ cells was not significantly correlated to the concentration of CD56^+^ cells in any of the analyzed groups: controls (*r* = 0.126; *p* = 0.75), uRPL-CD56^low^ (*r* = 0.361; *p* = 0.14), uRPL-CD56^Normal^ (*r* = 0.172; *p* = 0.40), and uRPL-CD56^high^ (*r* = −0.211; *p* = 0.45).

Analysis of the CD16/CD56 ratio demonstrated that the number of CD16^+^ cells was around 5–25% of the concentration of CD56^+^ cells in control endometria. In the uRPL-CD56^Normal^ subgroup, 59% had CD16/CD56 ratios similar to that of the control group. In 30% of cases from this subgroup, the percentage of CD16^+^ cells was increased, and in 11%, the number of CD16^+^ cells surpassed that of CD56^+^ cells (*p* < 0.001 vs. control). In 28% of the uRPL-CD56^low^ subgroup, CD16^+^ cells were elevated, and in 56%, they exceeded CD56^+^ cells. Only 6% of the uRPL-CD56^low^ subgroup had CD16/CD56 ratios corresponding to the control (*p* < 001 vs. control). The uRPL-CD56^high^ subgroup displayed CD16/CD56 ratio values comparable to controls (*p* = 0.92 vs. control). CD16/CD56 ratio values from the uRPL subgroups were different from each other (uRPL-CD56^low^ vs. uRPL-CD56^Normal^ *p* = 0.002; uRPL-CD56^low^ vs. uRPL-CD56^high^ *p* < 0.001; and uRPL-CD56^Normal^ vs. uRPL-CD56^high^ *p* < 0.001; Figure 6).

### 3.7. CD57/CD56 Correlation and Ratio

The number of CD57^+^ cells was not correlated with the concentration of CD56^+^ cells in the control (*r* = 0.066; *p* = 0.87) as well as in the uRPL-CD56^low^ (*r* = 0.070; *p* = 0.78) and uRPL-CD56^Normal^ groups (*r* = 0.112; *p* = 0.59). The uRPL-CD56^high^ group showed a negative correlation between CD57^+^ and CD56^+^ cells (*r* = −0.514; *p* = 0.04).

In order to identify differences between the control and uRPL subgroups, the CD57/CD56 ratio values were classified as follows: <20% (low), 21–60% (intermediate), and >60% (high). In the control group, 11% had low ratios of CD57^+^ cells, 67% had intermediate values, and 22% presented high ratios of CD57^+^ cells. Compared to the control, the uRPL-CD56^low^ subgroup showed 17% of cases with intermediate and 63% with elevated CD57/CD56 ratios (*p* = 0.03 vs. control). The uRPL-CD56^Normal^ subgroup presented similar CD57/CD56 ratios to those of the control group (*p* = 0.84 vs. control). In the uRPL-CD56^high^ subgroup, 75% showed low CD57/CD56 ratios whereas 25% had intermediate values in relation to the control (*p* = 0.02 vs. control). The uRPL subgroups had distinct CD57/CD56 ratios between each other (uRPL-CD56^low^ vs. uRPL-CD56^Normal^ *p* = 0.003; uRPL-CD56^low^ vs. uRPL-CD56^high^ *p* < 0.001; and uRPL-CD56^Normal^ vs. uRPL-CD56^high^ *p* = 0.003; Figure 6).

### 3.8. CD16/CD57 Correlation and Ratio

No significant correlations were found between the number of CD16^+^ and CD57^+^ cells in the control (*r* = −0.042; *p* = 0.92), uRPL-CD56^low^ (*r* = 0.384 *p* = 0.12), uRPL-CD56^Normal^ (*r* = 0.166 *p* = 0.42), or uRPL-CD56^high^ groups (*r* = 0.258 *p* = 0.33).

Analysis of the CD16/CD57 ratio demonstrated a predominance of CD57^+^ cells in 33% of control endometria (<100%). In 56%, the CD16/CD57 ratio were slightly increased towards CD16^+^ cells (100–200%). One control endometrium (11%) showed a higher ratio of CD16^+^ than CD57^+^ cells (>200%). The uRPL-CD56^low^ (*p* = 0.14 vs. control), uRPL-CD56^Normal^ (*p* = 0.05 vs. control), and uRPL-CD56^high^ subgroups (*p* = 0.01 vs. control) had respectively 6, 4, and 0% of cases with ratios indicating more CD57^+^ than CD16^+^ cells. The same subgroups presented 33, 42, and 25% of samples with slightly increased numbers of CD16^+^ cells compared to CD57^+^ cells. In 61% of uRPL-CD56^low^, 54% of uRPL-CD56^Normal^, and 75% of uRPL-CD56^high^, CD16^+^ cells outnumbered CD57^+^ cells to a great extent. No statistically significant differences were detected between the CD16/CD57 ratios compared to the uRPL subgroups (uRPL-CD56^low^ vs. uRPL-CD56^Normal^ *p* = 0.95; uRPL-CD56^low^ vs. uRPL-CD56^high^ *p* < 0.30; and uRPL-CD56^Normal^ vs. uRPL-CD56^high^ *p* = 0.29; Figure 6).

## 4. Discussion

The human endometrium contains a rich and dynamic assortment of immune cell populations. Compelling evidence links uRPL and other fertility disorders with impaired endometrial immune functions [7,40]. Nevertheless, evidence for [12,49] and against [50,51,52] the association of uNK cell levels with uRPL coexist. These conflicting results may arise from a broader spectrum of underlying immune disorders than usually identified in clinical and experimental settings. To better represent this complexity, the evaluation of uNK cells in endometrial biopsies should include additional markers to assess their cytotoxic and functional status. Our data demonstrate a high diversity of immunophenotypes in uRPL patients when CD45, CD56, CD16, and CD57 were evaluated. The ratios of uNK cells over total leukocytes (CD56/CD45) and highly cytotoxic subtypes (CD16/CD56 and CD57/CD56) were differently expressed depending on uNK cell concentration in the endometrium. 

As previously reported by our group and others, the endometrium of uRPL patients was characterized by cases with either low, normal, or high uNK cell counts [12,33]. These results reinforce the existence of a favorable uNK cell concentration range in fertile women. Furthermore, the occurrence of distinct phenotypes in RPL patients with low and high uNK cell counts indicates distinct pathogenic mechanisms. Whether these alterations are intrinsic to endogenous immune dysfunctions or are caused by exogenous factors (e.g., altered microbiota and pathogens) remains to be elucidated. Expansion of CD56^+^ uNK cells occurs in inflammatory conditions and immune reactions against bacterial components [53,54]. The influence of chronic inflammation/endometritis was ruled out in our study by the absence or low quantity (<3 cell/10 mm^2^) of CD138^+^ plasma cells in the endometrial samples. Increased levels of inflammatory mediators in the endometrium of infertile patients, including IL-12, IL-15, IL-18, and IL-27 [55,56,57], have the potential to influence uNK cell responses and to attract peripheral NK cells [58,59]. A positive correlation has been detected between uNK cell number and IL-15 expression in the endometrial stroma of RIF patients [54]. Evaluation of cytokine levels based on uNK ranges may help to clarify the influence of the endometrial milieu on uNK cell profiles in uRPL. 

Analysis of the CD56/CD45 ratio revealed that uNK cells corresponded to around 30 to 70% of the endometrial leukocytes in fertile women. In most samples from the uRPL-CD56^high^ subgroup, elevated numbers of CD56^+^ uNK cells, representing up to 90% of the endometrial immune cell population, accounted for high values of CD45^+^ cells. Nevertheless, some samples with high CD45^+^ cell concentrations displayed very low CD56^+^ cell counts. This can be an intrinsic characteristic of some cases of uRPL or may be due to an acute immune reaction or premature and excessive influx of leukocytes to the endometrium, as described in the transition of the luteal to premenstrual phases [60]. 

In an earlier publication, we defined <40 CD56^+^ cells/mm^2^ in the endometrium as cases with low numbers of uNK cells [12]. In the current study, based on a small cohort of fertile women as controls, we did not observe less than 90 cells/mm^2^. Furthermore, uRPL endometria with values below this limit had CD56/CD45, CD16/CD56, or CD57/CD56 ratios different from controls. These data encourage us to propose a stricter cutoff at 90 cells/mm^2^ to identify cases with low numbers of uNK cells which should be validated in a larger number of samples. Cutoff values may vary among different laboratories because of different immunohistochemical protocols (e.g., antibodies, amplification methods, and developers) and counting techniques (manual vs. automated) [61].

Several reports have shown that uNK cell cytotoxicity may create a hostile endometrial environment affecting embryo implantation and placentation [40]. Elevated levels of CD16^+^ and CD57^+^ cells were reported in the endometrium of RPL and RIF patients, pointing to a magnified cytotoxic potential of uNK cells in these conditions [10,11,36,37,38]. Our findings complement these previous studies by demonstrating that CD16^+^ and CD57^+^ cells have different relative levels in the uRPL endometrium depending on the concentration of CD56^+^ uNK cells. Furthermore, a predominance of CD16^+^ cells over CD57^+^ has been observed in uRPL patients. Of note, some patients classified as having normal numbers of uNK cells also present these cytotoxic markers upraised. 

Elevated levels of CD16^+^ and CD57^+^ cells were observed in around 40% of the uRPL-CD56^low^ subgroup. These results show that even cases with reduced numbers of uNK cells may have heightened cytotoxic potential. In the remaining 60%, CD16^+^ cells were more numerous than CD56^+^ cells, demonstrating a shortage of CD56^+^ uNK cells and the expression of CD16 by CD56^neg^ NK or other immune cells. Reduced numbers of uNK cells were reported in the endometrium of RPL patients infected with the human herpesvirus 6 (HHV6) [62]. The expansion of singular CD56^neg^CD16^+^ cells with altered functions can be promoted by viruses, including hepatitis C and cytomegalovirus (CMV) [63,64,65]. Differently from other subgroups, the uRPL-CD56^high^ subgroup presented a low proportion of CD57^+^ compared to CD56^+^ cells. Since CD57 is expressed by terminally differentiated cytotoxic NK cells [34,35], this result suggests impaired differentiation of such cells in some cases of uRPL.

Although the expressions of CD16 and CD57 indicate the cytotoxic potential of uNK cells, their effector functions are ultimately determined by the repertoire of activating and inhibitory receptors expressed at the cell surface, such as NKG2D, killer cell Ig-like receptors (KIR), and natural cytotoxicity receptors (NCR) [66,67]. A more in-depth characterization of uNK cell phenotype and functionality in distinct subgroups of uRPL patients will bring more information concerning the influence of these cells in infertility. 

Intravenous immunoglobulin (IVIg), intralipids, and glucocorticoids are some of the interventions employed in the treatment of RPL. Their efficacy, however, varies substantially among studies, precluding a consensus upon their effectiveness [3,68]. Lack of precise characterization of the endometrial immunological background may cause such variability. High numbers of uNK cells in endometrial biopsies constitute a parameter to recommend application of these immunomodulators. Our data highlight that not only the number of uNK cells but also the cytotoxic/immunomodulatory ratio should be taken into consideration to assess the uRPL endometrium. Better classification of the endometrial immunophenotypes in uRPL will aid in improving the definition of cases that may benefit from immunomodulatory treatment.

## 5. Conclusions

Our study reinforces a link between uRPL and disturbances in the endometrial immune cell profile. The data presented here highlight the relevance of a combined evaluation of CD45, CD56, CD16, CD57, and CD138 for identification of particular immunophenotypes in the endometrium of uRPL patients. The CD16/CD56 and CD57/CD56 ratios indicate endometrial cytotoxic/immunomodulatory balance. The CD56/CD45 ratio distinguishes cases with elevated leukocyte counts caused by elevated uNK cells from other conditions, and detection of CD138+ plasma cells indicates the presence of chronic endometritis.

Refinement of endometrial immune profiles will serve for more precise diagnostics in reproductive medicine and may help to develop personalized treatments for infertile women. 

## Figures and Tables

**Table 1:** List of antibodies used in the immunohistochemical analyzes.

Target	Species	Type	Supplier	Dilution
CD16	Mouse	Monoclonal—DJ130	Santa Cruz	1:200
CD45	Mouse	Monoclonal—2B11 + PD7/26	Dako	1:200
CD56	Mouse	Monoclonal—123C3	Dako	1:100
CD57	Mouse	Monoclonal—TB01	Dako	1:100
CD138	Mouse	Monoclonal—MI15	Dako	1:100

## Abbreviations

CMV	cytomegalovirus
DAB	3,3′-diaminobenzidine
HHV6	human herpes virus 6
IVig	intravenous immunoglobulin
KIR	killer cell Ig-like receptors
NCR	natural cytotoxicity receptors
MIP-1β	macrophage inflammatory protein 1β
NK	natural killer cells
uNK	uterine natural killer cells
NCAM	neural cell adhesion molecule
RIF	recurrent implantation failure
RPL	recurrent pregnancy loss
RT	room temperature
TBST	Tris-buffered saline-Tween20
uRPL	unexplained recurrent pregnancy loss
uRPL-CD56^high^	uRPL subgroup of high uNK cell counts
uRPL-CD56^low^	uRPL subgroup of low uNK cell counts
uRPL-CD56^normal^	uRPL subgroup of normal uNK cell counts
